# Computational Prediction of Host-Parasite Protein Interactions between *P. falciparum* and *H. sapiens*

**DOI:** 10.1371/journal.pone.0026960

**Published:** 2011-11-17

**Authors:** Stefan Wuchty

**Affiliations:** National Center for Biotechnology Information, National Institutes of Health (NIH), Bethesda, Maryland, United States of America; Kenya Medical Research Institute - Wellcome Trust Research Programme, Kenya

## Abstract

To obtain candidates of interactions between proteins of the malaria parasite *Plasmodium falciparum* and the human host, homologous and conserved interactions were inferred from various sources of interaction data. Such candidate interactions were assessed by applying a machine learning approach and further filtered according to expression and molecular characteristics, enabling involved proteins to indeed interact. The analysis of predicted interactions indicated that parasite proteins predominantly target central proteins to take control of a human host cell. Furthermore, parasite proteins utilized their protein repertoire in a combinatorial manner, providing a broad connection to host cellular processes. In particular, several prominent pathways of signaling and regulation proteins were predicted to interact with parasite chaperones. Such a result suggests an important role of remodeling proteins in the interaction interface between the human host and the parasite. Identification of such molecular strategies that allow the parasite to take control of the host has the potential to deepen our understanding of the parasite specific remodeling processes of the host cell and illuminate new avenues of disease intervention.

## Introduction

Currently, little is known about large-scale protein interactions between cells, although large-scale maps are an important foundation for the understanding of the ways pathogens interact, invade and seize control of their human hosts. Recently, Uetz et al. released the first small map of computationally inferred physical protein interactions between the human host, the Kaposi-Sarcoma associated Herpesvirus and the Varicella-Zoster-Virus [1]. In another approach, Calderwood et al. [2] experimentally constructed a map of physical protein interactions between the Epstein-Barr-Virus and the human host. Similarly, de Chassey et al. [3] generated a large-scale map of interactions between the Hepatitis C virus and the human host. Furthermore, interaction networks between the human immunodeficiency virus (HIV) and the human host have been investigated [4], [5], as well as co-factors that enable HIV and the influenza virus to infect a host cell [6], [7], [8]. In addition, Dyer et al. compared experimentally known interactions of different viruses with the human host [9].

Recently, Vignali et al. released the first small map of experimentally determined protein interactions between the human host and the malaria parasite *Plasmodium falciparum* [10], a study that was preceded by computational predictions of host-parasite interactions [11], [12]. In particular, Davis et al. [12] comparatively inferred protein-protein interactions between human host cells and several pathogens utilizing structural protein data.

Based on large-scale sets of species-specific protein interactions in *H. sapiens* and the malaria parasite *P. falciparum* I inferred potentially conserved host-parasite interactions by utilizing orthologous protein groups. Furthermore, experimentally determined host-parasite interactions were used to generate potential interaction candidates by searching for organism-specific homologous proteins. To mitigate the potential influence of false-positive interactions, I applied a machine learning approach to assess the quality of the predicted interactions. Subsequently, predicted interactions between the human host and the parasite were filtered, accounting for parasite protein specific characteristics that are conducive to potential host-parasite interactions. In addition, I demanded that both parasite and human proteins were co-expressed in the corresponding parasitic cell cycle stages and human tissues/cells.

The combination of predicted interactions with experimentally determined and structurally inferred interactions allowed for a large set of potential interactions between proteins of the malaria parasite *P. falciparum* and the human host. In comparison, the separate and combined sets of interactions shared similar characteristics. In particular, the pathogen seemed to utilize its protein repertoire in a combinatorial way by predominately targeting hub proteins. Such a strategy probably allows the parasite to take control of the human host cell, effectively reaching into signaling and other cellular functions of the host cell. Several prominent pathways of signaling and regulation proteins were predicted to interact with parasite chaperones, suggesting an important role of such proteins in the interaction interface between the human host and the parasite.

## Results

**Figure 1:**
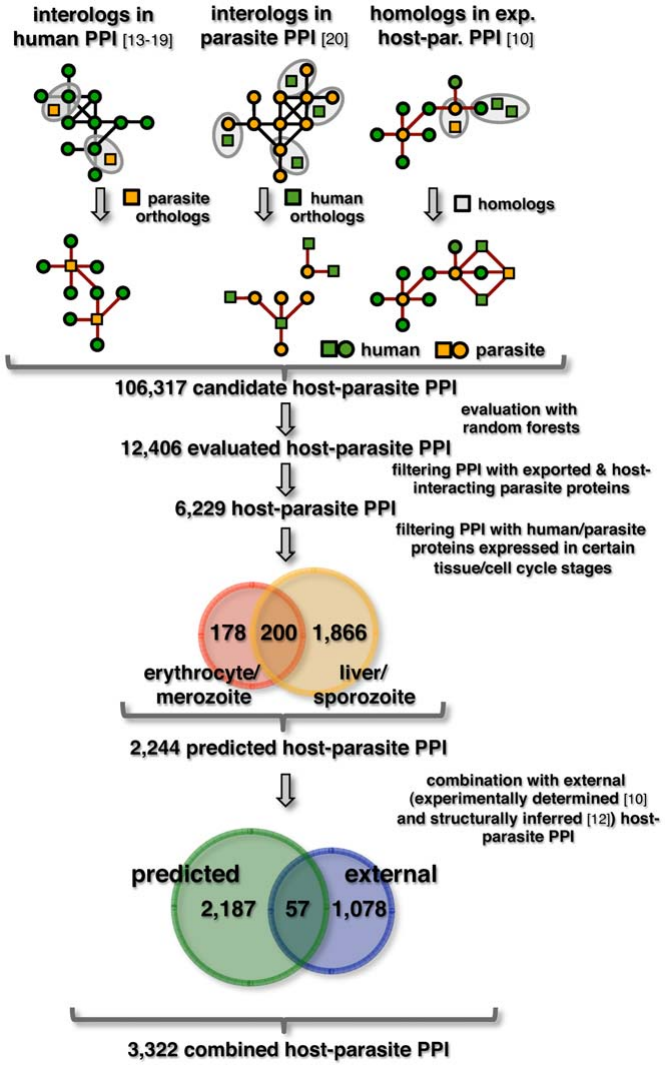
Procedure to determine interactions between human host and parasite proteins. A candidate interaction was identified in the web of human interactions if an interacting human protein had an ortholog in *P. falciparum*. Analogously, a potential interaction occurred if a protein in the parasite interaction network had a human ortholog. In a set of experimentally determined host-parasite interactions a host-parasite interaction was found, if the interacting proteins had homologs in the corresponding organism. The combination of all sources provided a total of 106,317 candidate interactions. The quality of the predicted interactions was assessed using the random forest algorithm, a machine learning method that classifies interactions as a function of the corresponding protein's sequences. Subsequently, 12,406 interactions thus obtained were filtered if they involved parasite proteins that were exported or carried molecular characteristics, enabling them to interact with the human host. While this step provided 6,229 interactions only host-parasite interactions were accounted for that occurred between proteins expressed in the parasitic merozoite stage and human red blood cell as well as in the sporozoite stage and liver cells. Accordingly, partially overlapping sets of 378 and 2,044 interactions were obtained, pooling a total of 2,244 predicted host-parasite protein interactions. In the last step predicted interactions were combined with external data such as structurally inferred and experimentally obtained interactions, providing a set of 3,322 combined host-parasite interactions.

Exploring a sequence orthology/homology based approach to determine protein-protein interactions between human host and parasite proteins, a flowchart of the procedure is shown in Fig. 1.

### Inferring Interactions between *H. sapiens* and *P. falciparum*

I assembled a network of 93,178 interactions between proteins of *H. sapiens* using large-scale high-throughput screens [13], [14], [15] and several interaction databases [16], [17], [18], [19]. In addition, a web of 2,743 experimentally determined interactions [20] in *P. falciparum* was utilized as well. Compiling 2,664 orthologous pairs of human and parasite proteins from the InParanoid database [21], a candidate interaction was found in the web of human interactions if a protein had a parasite ortholog. Analogously, a potential interaction was detected if a parasite protein in the parasite interaction network had a human ortholog. Similarly, I utilized 444 experimentally determined interactions between human and parasite proteins [10]. To identify candidate interactions, a BLAST search was performed to find homologs of interacting parasite and human proteins. Specifically, I considered a pair of proteins homologous if their E-value was <10^−6^. Combining all sources, the final set was composed of 106,317 candidate interactions between 2,096 parasite and 8,650 human proteins.

### Quality Assessment of Candidate Interactions

**Figure 2:**
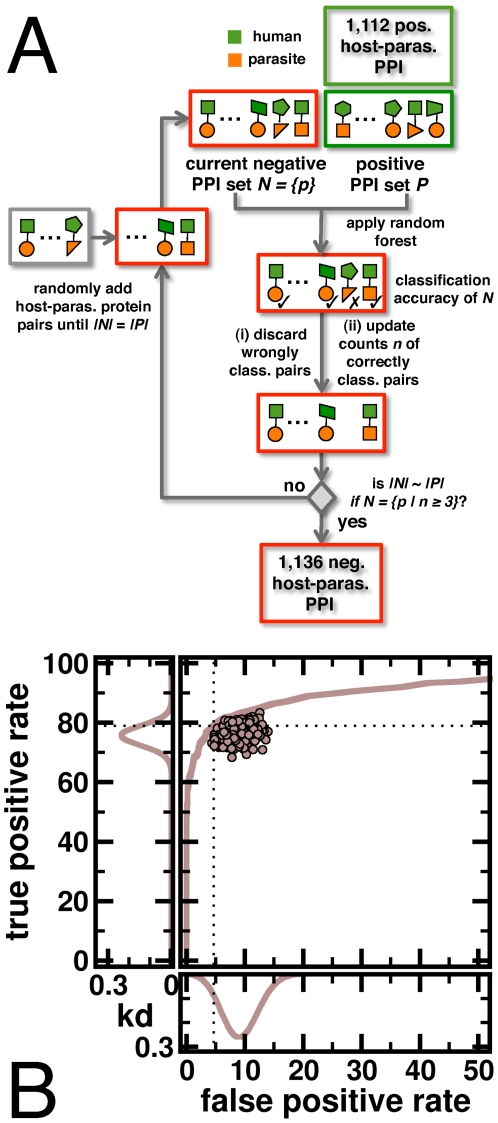
Determination of a negative interactions set. (**A**) As a positive training set I utilized 1,112 interactions between the human host and the parasite *P. falciparum* that have been previously inferred from protein structures. A non-interacting training set of equal size was constructed by randomly sampling pairs of human and parasite proteins that did not appear in the positive training set. Applying the random forest algorithm pairs of proteins that were incorrectly classified as interacting were discarded, and counts of correctly classified pairs were updated. If the number of pairs in the negative set that were sampled at least 3 times was roughly the size of the positive training set, the procedure terminated. Otherwise, the negative set of was filled with randomly sampled protein pairs until positive and negative training sets had the same size again. Previously described steps were repeated until the procedure finally terminated, providing a negative set of 1,136 non-interacting pairs. (**B**) Applying the random forest algorithm the training sets allowed for a true positive rate TPR = 78.9% and a false positive rate FPR = 4.7% (dashed lines) in a pronounced ROC curve. In a test-retest analysis, the classifier was trained on ⅔^rd^ of randomly picked training data. Its performance was tested on the remaining ⅓^rd^, indicating that the proposed method was largely robust to noise.

While sequence searches provided a large sample of candidates, such interactions most certainly contained a considerable amount of false positives. Assessing the quality of interactions I used the random forest algorithm [22] to consider interacting candidate pairs as a function of their sequence composition. Grouping amino acids in 7 classes [23] the frequency of each triple combination of classes over all amino acid triplets in each protein was determined. An interaction between a protein pair was represented by a 343-dimensional vector where each vector unit held the frequency difference of a given class combination. As a positive training set 1,112 structurally inferred host-parasite interactions [12] were utilized. In the absence of a comprehensive negative training set of non-interacting protein pairs a simple heuristic was applied (Fig. 2A): As a negative, non-interacting training set of equal size, protein pairs that did not appear in the positive training set were randomly sampled. Using such sets the random forest algorithm was applied, allowing cross-validation by reporting the fraction of times protein pairs were correctly classified. While wrongly classified pairs were discarded, the counts of correctly classified pairs were updated. This process was repeated until a negative set of roughly the same size of the positive training set was obtained. In addition, each non-interacting pair needed to be sampled at least 3 times, providing 1,136 non-interacting pairs (Fig. 2A). Both sets of interactions are available in Tables S1 and S2.

Using these sets as positive and negative training sets the random forest algorithm was applied to check the quality of candidate interactions. In particular, the algorithm provided the fraction of decision trees that reported a pair of proteins as interacting. Assuming different fractions as classification thresholds a ROC curve in Fig. 2B was constructed. Defining an interaction when half of all decision trees voted that way the approach allowed for a false positive rate FPR = 4.7% and a true positive rate TPR = 78.9%. To assess the reliability of the classification approach a test-retest analysis was performed by training the random forest algorithm with ⅔^rd^ of randomly picked training data. Subsequently, the corresponding true positive and false positive rates of the classification results were determined using the remaining ⅓^rd^ of data. Repeating such random trials 1,000 times dot clouds in Fig. 2B indicated that the method was largely robust in the presence of random noise.

Assessing the quality of candidate interactions with the random forest approach 12,406 interactions between 1,007 parasite and 3,614 human proteins were finally obtained.

### Functional and Expression Filtering of Host-Parasite Interactions

Putatively, only a subset of involved parasite proteins carried molecular characteristics that allowed interactions with the human host, prompting me to compile a list of 1,302 parasite proteins with such molecular features (Table S3, for details please see Materials and Methods). Filtering candidate interactions that involved such parasite proteins provided 6,229 links between 322 parasite and 2,535 human proteins.

In addition, host-parasite interactions also were more likely to appear between human proteins that were expressed in liver tissue [24] and parasite proteins appearing in the sporozoite stage of the parasite's cell cycle [25] when the parasite invades human liver cells. Interactions were analogously filtered if they involved proteins that were expressed in the erythrocyte and the merozoite stage, respectively. While these constraints provided 2,066 and 378 interactions, respectively, a small set of 200 host-parasite interactions were shared. Combining both sets, a total of 2,244 interactions involved 128 parasite and 1,389 human proteins.

In the last step, the set of predicted interactions was combined with external data, such as experimentally determined and structurally inferred interactions, providing a total of 3,322 interactions between 388 parasite and 1,886 human proteins (Table S4).

### Statistical Properties of Predicted Interactions

**Figure 3:**
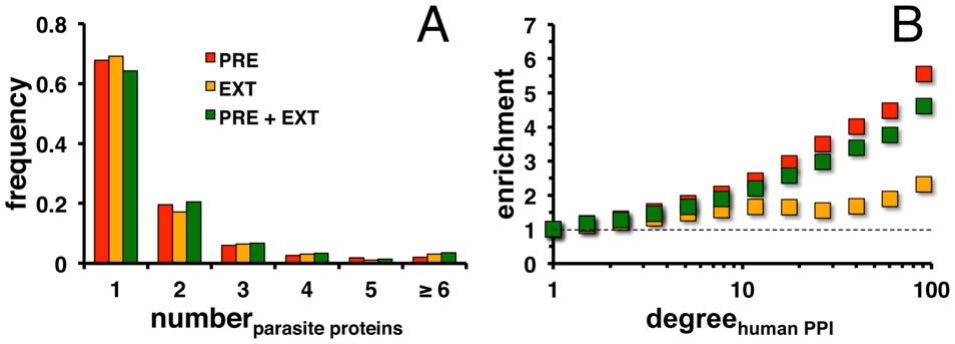
Characteristics of predicted and external interactions. (**A**) In the set of predicted interactions (PRE) a majority of host proteins interacted with a low number of pathogen proteins and vice versa. Such a result was confirmed by the set of external interaction data (EXT) as well as the combined set of host-parasite interactions (PRE+EXT). (**B**) Utilizing a network of human protein-protein interactions the enrichment of targeted human host proteins as a function of their number of interaction partners was determined. Considering all three sets of host-pathogen interactions separately highly connected host proteins appeared to be prime targets of the parasite.

Utilizing predicted interactions between proteins of *P. falciparum* and the human host the number of parasite proteins that targeted a host protein was calculated. Fig. 3A indicates that a majority of host proteins interacted with a low number of pathogen proteins and *vice versa*, suggesting that many proteins were targeted by the parasite in a combinatorial way. External data such as experimentally determined and structurally inferred interactions and their combination with predicted interactions confirmed our initial observation.

Considering topological integrity, the bipartite network of predicted interactions between parasite and host proteins was composed of 18 disconnected subnetworks. Notably, the largest subnetwork contained the vast majority of parasite and host proteins. By comparison, a bipartite network of external interactions was composed of 106 subnetworks, while a combined network broke into 62 components. If the choice of targeted host proteins were a random process a randomly assembled bipartite network would break into many more disconnected parts. In a null-model, parasite proteins were connected to the same number of randomly picked human proteins that were expressed in the erythrocyte and liver. Repeating these steps 10,000 times randomized networks significantly broke into a larger number of components than observed (P<10^−4^). Such a result indicates that the integrity of the predicted host-parasite interaction network was the result of a significantly non-random process.

Since they are important proteins highly connected hubs may be prime targets of parasite proteins. Utilizing a previously described network of human interactions, human proteins were assigned to groups that had at least a certain number of interaction partners in the human interaction network. Specifically, the fraction of human proteins that were targeted by parasite proteins was calculated and compared to the corresponding fraction of proteins that were randomly picked as parasite targets out of all proteins in the human interaction network. If there existed no correlation to the number of interactions in a human protein interaction network, the ratio of these fractions was expected to be 1 in all groups. However, Fig. 3B showed that especially highly connected proteins were more affected by the parasite, using predicted interactions. While the sets of external and combined host-parasite interactions recovered this result as well, they showed weaker trends.

### Functional and Statistical Implications of Targeted Host Proteins and Signaling Pathways

Considering the combined set of host-parasite interactions, overrepresented GO terms of biological processes in the pool of interacting host proteins were determined. Utilizing GOStat [26] many processes annotated as signaling and regulation processes were observed as being enriched, indicating that the parasite uses its protein repertoire to influence important signaling and regulation processes (Table S5). Such results called for an investigation of signaling pathways that potentially are another level of systems information the parasite exploits to control the host cell.

**Figure 4:**
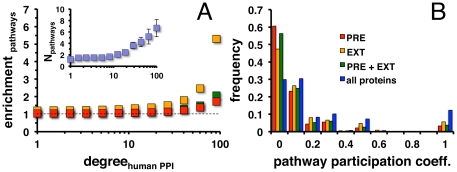
Pathway specific characteristics of predicted and external interactions. (**A**) In a network of human protein interactions the mean number of pathways a given human protein is involved in increased with the number of interaction partners (inset). The enrichment of such targeted pathways was determined as a function of their number of interaction partners. Utilizing predicted (PRE), external (EXT) and both sets (PRE+EXT) of host-parasite interactions, highly connected, targeted proteins appeared to be increasingly involved in such pathways. (**B**) A low value of the pathway participation coefficient indicated that the interaction partners of a protein in a human protein interaction network reached many different pathways and vice versa. Considering all human proteins that were involved in pathways, the majority of proteins have low pathway participation coefficients. Considering targeted proteins in all sets of host-parasite interactions the initially observed trend was significantly reinforced in all cases (Wilcoxon rank-sum test, P<10^−10^).

The analysis relied on the strength of 184 manually curated signaling pathways from the Pathway Interaction Database PID [27]. In a human protein interaction network highly connected proteins appeared in an increasing number of signaling pathways (inset, Figure 4A). Such an observation emphasized the role of protein hubs being involved in numerous signaling pathways, suggesting that the parasite has taken advantage of the host network at the pathway level as well. In the previously described human interaction network the number of different pathways was determined that involved targeted proteins with a certain number of interaction partners. In a null-model targeted proteins were randomly picked out of all proteins in the human protein interaction network. Determining the number of different pathways involving such randomly targeted proteins, the fraction of such sets of pathways was calculated. Separate and combined sets of predicted and external host-parasite interactions indicated that that highly connected, targeted proteins appeared in an increasing number of pathways with higher degree (Fig. 4A).

As a further hypothesis, a parasite may effectively mediate the infection while ensuring variety given the tendency to target signaling pathway hubs. As a measure of diversity, the pathway participation coefficient (PPC) was defined: if a given protein predominantly interacted with partners that were members of the same pathway, PPC tends toward 1, while the opposite holds if the interaction partners of the considered protein were distributed among many different pathways. Fig. 4B indicated that human proteins largely reached a variety of pathways through its interaction partners in a human protein interaction network. However, predicted, external and combined interactions showed significantly enforced maxima around low values of the pathway participation coefficient in Fig. 4B (P<10^−10^, Wilcoxon rank-sum test). Such observations strongly indicated that targeted human proteins effectively secured the parasite's reach into a breadth of signaling activities without inundating any particular one.

**Figure 5:**
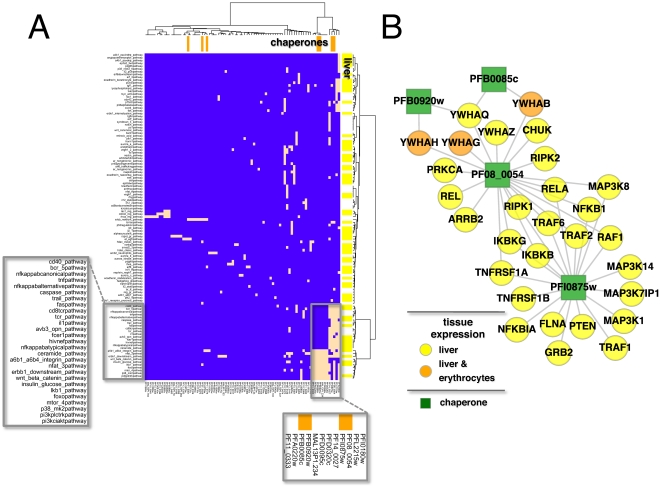
Involvement of host-parasite interactions in signaling pathways. (**A**) In a bipartite matrix a parasite protein was linked to a pathway if the corresponding targeted proteins were enriched in the given pathway (FDR<0.05, Fisher's exact test). Utilizing the combined set of host-parasite interactions the matrix was composed of 83 parasite proteins that interacted with proteins in 117 pathways. In addition, a large fraction of pathways had >95% of their proteins being expressed in liver. Specifically, a cluster of prominent signaling pathways emerged that were largely under the control of parasite specific chaperone proteins. In (**B**) all interactions between parasite chaperones and host proteins that appeared in the cluster of pathways were mapped. Significantly, such a network revolves around parasite proteins PF08_0054 and PFI0875w that garnered most of the host-parasite interactions and largely interfered with proteins of the TNF pathway.

To obtain a detailed functional view of the parasite's intervention points a bipartite matrix between parasite proteins and signaling pathways was constructed. Specifically, a parasite protein was connected to a pathway, if the corresponding targeted proteins were enriched in the given pathway using a Fisher's exact test (FDR<0.05 [28]). Utilizing the combined set of host-parasite interactions 83 parasite proteins emerged that interacted with 117 pathways (Fig. 5A). Pathways were checked if their proteins were expressed in liver and red blood cells. Demanding that more than 95% of proteins were expressed, I found no such pathways in the red blood cell because of its small proteome. In turn, however, a large fraction of pathways were expressed in liver. Out of 95 parasite chaperones [29] 7 significantly appeared in the set of 83 parasite proteins (hypergeometric test, P<10^−30^). Ward clustering of the matrix indicated a group of signaling pathways that were linked to 4 parasitic chaperone proteins (box, Fig. 5A). Notably, parasite chaperones PF08_0054 and PFI0875w seemed to play an important role in interfering with signaling pathways since both proteins reached a large number of different pathways. Host-parasite interactions between the indicated 4 parasite chaperones and host proteins that appeared in the corresponding, connected signaling pathways were indeed dominated by PF08_0054 and PFI0875w by co-interacting with many prominent regulation and signaling proteins (Fig. 5B). Notably, both chaperones strongly interacted with members of the TNF pathway [30]. Specifically, the chaperones targeted members of the TNF receptor associated factor (TRAF) protein family as well as of the receptor-interacting protein (RIPK) family of serine/threonine protein kinases. In the TNF pathway, TRAF proteins recruit protein kinase IKK that is activated by RIPK proteins. IKK phosphorylates the inhibitory IκBα (NFKBIA), leading to its degradation of IκBα and the release of NF-κB, potentially triggering an inflammatory response. Both NFKBIA and subunits of NF-κB (NFKB1, NFKB2) interacted with both PFI0875w and PF08_0054. Targeted by the same parasite chaperones, members of the Mitogen-activated protein kinase kinase kinase, MAP3K8 and MAP3K14, are able to alternatively induce the production of NF-κB [31], [32]. The observation that the parasite massively interacted with proteins of the TNF- and NF-κB pathways may indicate the objective to curb an inflammatory response that may ensue after invading a cell. Notably, the parasite may achieve such a goal through the usage of molecular remodeling function.

## Discussion

Significant challenges currently impair experiments to develop large-scale empirical maps of interactions between the human host and the malaria parasite *P. falciparum*, prompting the implementation of a sequence homology approach to suggest putative host-parasite protein interactions. Although sequence homology is a powerful technique, large inserts obscure homology signals in gene/protein sequences of *P. falciparum* that might hamper the detection of orthologs in different organisms. To widely mitigate these effects, predictions were checked using a machine learning method that was trained on structurally inferred interactions between human and parasite protein interactions.

To ensure utmost biological relevance, predicted interactions were further filtered that involved parasite proteins with parasite specific characteristics, making the underlying parasite protein conducive to interact with the human host. Accounting for expression characteristics interactions were further filtered when their host and parasite proteins were expressed in the corresponding human tissues and parasite specific cell cycle stages.

Sets of predicted and external interactions such as experimentally obtained [10] and structurally inferred interactions [12] showed a low overlap while experimental and structurally obtained interactions had no interactions in common. Despite these differences, topological observations indicated shared characteristics. Specifically, degree distributions of all sets of host-parasite interactions highlighted a small number of human proteins that interacted with many parasite proteins. Such result suggested that the parasite utilizes its proteomic repertoire in a combinatorial way, an observation that is known from other pathogens [33] as well. The subtle structure of the human interactome reveals sites that are not only topologically important on their own, but also represent significant pathogen targets. Previously, network analyses indicated that hubs are important for maintaining the integrity of a network [34]. While random attacks hardly hit such highly connected nodes, networks break easily into disconnected parts when hubs are attacked. Proteomic equivalents potentially allow the parasite to reach into functional pathways, probably facilitating a diverse, but focused interaction. Functional and topological promiscuity of hub proteins in pathways might enable the host to maintain a complex system with relatively few proteins. Tapping this feature in an economic yet effective way the malaria parasite uses combinations of pathogen proteins to interact with a variety of different pathways, allowing its survival and control of the human host cell.

Untangling the intricate web of intertwined pathways is essential to thoroughly understand the pathogenesis of the parasite. In the light of these observations, targeted proteins that are shared by a large number of relevant pathways may be key players in subtle molecular strategies to seize control of a host cell. In addition, the analysis of targeted host proteins that appear in many signaling pathways may point to molecular sites that could be exploited to limit a parasite in a systematic way.

A potential role in the parasite specific interference in signaling pathways was indicated by the involvement of parasite chaperone proteins. In particular, chaperones were significantly present interacting with human proteins that play important roles in cell signaling. While the fundamentally important role of chaperones for the inner workings of the parasite cell has been recently indicated [35], [36], an involvement of chaperones in host-parasite interactions has been suggested as well [37]. Since they mediate the (un-)folding and (dis-)assembly of other macromolecular structures, the parasite chaperones might remodel protein structures in the host cell in ways that have considerable downstream effects, helping the parasite to take control of the cell. Such results can serve as testable hypotheses especially relating to *P. falciparum* biology for which focused experimentation might not only increase our understanding of patterns of host-parasite interactions. Such webs of well-defined host-parasite interactions can also serve as maps of parasitic interference that suggest avenues guiding future efforts to eradicate malaria, a disease that despite large efforts still plagues human kind.

## Materials and Methods

### Parasite Interacting Proteins

An initial list of 1,015 proteins in *P. falciparum* with molecular characteristics that facilitated a likely interaction with human host proteins was compiled. In particular, parasite proteins with a host cell-targeting signal, allowing proteins to cross into the human host cell by passing several membranes [38], [39], [40] were selected as well as proteins that had trans-membrane domains, signaling peptides and/or were located in the red blood cell membrane [25]. This set was augmented with 287 proteins that were implicated in host-pathogen interactions, pathogenesis, defense response, placed in the host cell as suggested by their corresponding GO annotation or PlasmoDraft prediction [41], [42], [43] as well as were known to interact experimentally [10].

### Host-Parasite Interactions

As a source of experimental protein interactions between the human host and the malaria parasite *P. falciparum* 444 experimentally determined interactions were utilized [10], where human proteins were represented by their gene symbols. Furthermore, 1,012 structurally inferred host-parasite interactions were used [12] where human proteins were indicated by their ENSEMBL protein symbols [44]. Using gene symbols, this set broke down to 691 interactions.

### Human Protein Interactions and Molecular Pathways

Human protein-protein interaction data were collected from large-scale high-throughput screens [13], [14], [15] and several interaction databases [16], [17], [18], [19], totaling 93,178 interactions among 11,691 proteins annotated by their gene symbols. Human signaling pathway information was retrieved from the NCI/NIH/Nature Pathway Interaction Database (PID) [45] providing information about 184 different human signaling pathways.

### Expression Data

From a mass-spectroscopic proteome analysis of the human red blood cell [46] 1,578 human proteins were selected. In addition, a set of 19,031 human proteins that were expressed in human liver tissue was utilized [24]. Determined with mass-spectroscopic methods, 838 proteins predominantly expressed in the parasitic merozoit stage were selected while 1,038 parasite proteins detected in the sporozoit stage [25] were used.

### Orthologous Proteins

Utilizing all-versus-all BLASTP searches determined by the InParanoid script [21] in protein sets of two species, sequence pairs with mutually best scores were selected as central orthologous pairs. In order to enhance the quality, only BLAST matches with a score >40 bits and ≥50% coverage of the longer sequence were accounted for. Proteins of both species that showed such an elevated degree of homology were clustered around these central pairs, a procedure that formed orthologous groups. The quality of the clustering was further assessed by a standard bootstrap procedure. Accounting for pairs with 100% probability, InParanoid provided a set of 2,664 orthologous protein pairs in *P. falciparum* and *H. sapiens*.

### Mapping Host-Parasite Protein Interactions as Vectors of Amino Acid Triplets

Here, two proteins were determined to interact as a function of the sequence composition of the protein pair. Grouping amino acids in 7 classes [23] the frequency of all 7×7×7 = 343 combinations of such classes in a protein was calculated. Specifically, the frequency of a combination *k* over all 343 class combinations in a protein *i* of a given interaction was defined as, 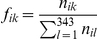, where *n_ik_* was the occurrence of combination *k* in protein *i*, scanning over all consecutive amino-acid triplets. An interaction between a parasite protein *i* and a host protein *j* was represented by a 343-dimensional vector where each vector unit held the frequency difference of a combination *k*,. 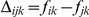


### Random Forest Classification

Random Forests is an ensemble learning method [22] where each decision tree is constructed using a different bootstrap sample of the data (‘bagging’). In addition, random forests change how decision trees are constructed by splitting each node, using the best among a subset of predictors randomly chosen at that node (‘boosting’). Compared to many other classifiers, this strategy turned out to perform very well and was robust against over-fitting. In more detail, classification performed by random forests is based on three steps: (i) *N* bootstrap samples are drawn from the underlying data. For each of the bootstrap samples, an un-pruned decision tree is constructed where at each node *M* predictors are randomly sampled, (ii) the best split from those variables is finally picked, and (iii) new data is predicted by aggregating the predictions of *N* trees.

For each of 1,000 decision trees 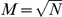 variables out of all *N* = 343 triplet combinations and ⅔^rd^ of all protein pairs were sampled. Since each interaction therefore ends up in the remaining ⅓^rd^ several times (*i.e.* out-of-bag examples), the random forest algorithm reports the fraction of times the pair of proteins was classified as interacting. Specifically, an interaction was found if more than half of the decision trees voted that way (*i.e.* fraction *f*>0.5).

### ROC Curve

Using out-of-bag examples as a cross-validation set a ROC curve was constructed by defining true positive (*TP*), true negative (*TN*), false positive (*FP*) and false negative hits (*FN*). Furthermore, true positive and false negative rates were defined as 
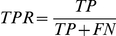
 and, 
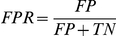
, respectively.

### Enrichment of Targeted Proteins and Pathways

Proteins were grouped according to their number of interactions in a given human protein interactions network. Each group was represented by 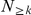 proteins that had at least a certain number of interaction partners, *k*. In each group the number of proteins that were targeted by the parasite, 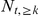 , was calculated. Randomly picking targeted protein out of all proteins in the given human protein interaction network, 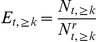 was defined as the enrichment of targeted proteins where 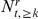 was the random number of targeted proteins among all 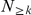 proteins.

In the same groups of human proteins, 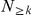 the set of different pathways that involved targeted proteins, , 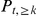, was determined. Randomly picking targeted proteins out of the set of all proteins that appeared in considered pathways, 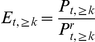 was defined as the enrichment of pathways, where 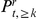 was the set of pathways randomly targeted proteins were involved in.

After averaging *E* over 10,000 randomizations *E>1* pointed to an enrichment and *vice versa* [47], while *E = 1* indicated a random process.

### Pathway Participation Coefficient

For each protein *i* that was involved in pathways and a human protein interaction network, the corresponding pathway participation coefficient *PPC* in the total set of pathways *P* was defined as 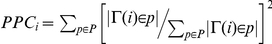, where 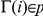 was the set of interaction partners of *i* that appeared in the same pathway *p*. The definition of the pathway participation coefficient resembled the Simpson diversity, a measure to quantify biodiversity in a habitat [48]. Specifically, Simpson diversity was defined as 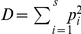, where *p_i_* was the fraction of all organisms that belonged to species *i*. Equivalently, *PPC* can range from 0 to 1, and *PPC* tend to 1 if a protein predominantly interacted with partners that were members of the same pathway. In turn, *PPC* tend to 0 if interaction partners of a given protein were distributed among many different pathways [33].

## Supporting Information

Table S1 **Positive training set including 1,112 interactions.**
(XLS)

Table S2 **Negative training set including 1,136 interactions.**
(XLS)

Table S3 **1,302 annotated parasite proteins that carry molecular characteristics, allowing them to interact with the human host.**
(XLS)

Table S4 **Combined set of 3,322 interactions including predicted (PRE) and external (EXT) interaction data.**
(XLS)

Table S5 **100 most enriched GO biological processes of host proteins that interact with parasite proteins in the combined set of host-parasite interactions.**
(XLS)
